# 0438. Effects of positive end-expiratory pressure (PEEP) on the pattern of breathing during neurally adjusted ventilatory assist. A pilot study in a mild ards porcine model

**DOI:** 10.1186/2197-425X-2-S1-P28

**Published:** 2014-09-26

**Authors:** M Pellegrini, G Perchiazzi, A Ronéus, I Andersson, T Fiore, A Larsson, G Hedenstierna

**Affiliations:** Università di Bari Aldo Moro, Dept. of Emergency and Organ Transplant, Bari, Italy; Uppsala University, Dept. of Surgical Sciences, Uppsala, Sweden; Kalmar Hospital, Dept. of Anesthesia and Intensive Care, Kalmar, Sweden

## Introduction

Spontaneous breathing during mechanical ventilation is still controversial. It is well known that incongruous patterns of mechanical ventilation can trigger or exacerbate lung injury. NAVA is a mode of mechanical ventilation that delivers ventilatory assist in proportion to the Electrical activity of the Diaphragm (EDi), which mirrors the performance of the whole respiratory system. However during NAVA, the effects of PEEP on the EDi signal, respiratory rate and breathing heterogeneity are unknown.

## Objectives

The purpose of the present pilot study was to evaluate the effects of PEEP on the ventilatory pattern during NAVA support in a porcine model of mild ARDS.

## Methods

Three anesthetized, tracheostomized and spontaneous breathing piglets, underwent NAVA ventilation. Mild ARDS was achieved by repeated lung lavages and bronchial aspiration (targeting a PaO_2_/FiO_2_ ratio of 200). Each animal was ventilated with four different supports: Optimal NAVA (NAVA_OPT_, defined by using Brander et al. method^1^), Low NAVA (NAVA_LOW_ defined as NAVA_OPT_ -20%), High NAVA (NAVA_HI_ defined as NAVA_OPT_ +20%) and unsupported breathing (NAVA_zero_ defined as NAVA of 0 cmH_2_O/µV). During each pattern of ventilation, a stepwise change in PEEP was applied, first incrementing PEEP from 0 to 15 cmH_2_O in steps of 3 cmH_2_O; then decrementing it from 15 to 0 cmH_2_O with the same steps. During each step, after a steady state was reached, the main hemodynamic and respiratory parameters were recorded and a breath-by-breath analysis of flow, airway pressures and EDi waves, was performed by using custom made Matlab (The Mathworks, Natick, USA) codes. We defined as index of breathing pattern heterogeneity (BPH), the percentage of breaths whose maximum EDi (EDI_max_) exceeds by 2 standard deviations the mean of EDI_max_ (EDI_max, mean_); minimum EDi (EDI_min, mean_) is the mean of lowest EDIs reached in a breath sample. Statistical analysis was performed by testing significant differences of respiratory rate (RR), EDI_max, mean_ , EDI_min, mean_ and BPH at each applied PEEP using the Student's T test (α=0.05) at the various NAVA levels.

## Results

RR was inversely related to applied PEEP. Increasing PEEP, EDI_max, mean_ increases while EDI_min, mean_ decreases. Breathing pattern heterogeneity did not change in relation to PEEP variation.

## Conclusions

The application of PEEP during spontaneous breathing in a model of mild ARDS influenced the ventilatory pattern, i.e., the respiratory rate, the diaphragmatic tonic (EDI_min, mean_) and the phasic contraction (EDI_max, mean_). If these effects are valid in patients, they will be clinically important when using NAVA as a weaning mode.
